# A Genome-Scale Analysis of the *PIN* Gene Family Reveals Its Functions in Cotton Fiber Development

**DOI:** 10.3389/fpls.2017.00461

**Published:** 2017-03-30

**Authors:** Yuzhou Zhang, Peng He, Zuoren Yang, Gai Huang, Limin Wang, Chaoyou Pang, Hui Xiao, Peng Zhao, Jianing Yu, Guanghui Xiao

**Affiliations:** ^1^Key Laboratory of the Ministry of Education for Medicinal Plant Resources and Natural Pharmaceutical Chemistry, National Engineering Laboratory for Resource Development of Endangered Crude Drugs in the Northwest of China, College of Life Sciences, Shaanxi Normal UniversityXi’an, China; ^2^Institute for Advanced Studies/College of Life Sciences, Wuhan UniversityWuhan, China; ^3^State Key Laboratory of Cotton Biology, Cotton Research Institute – Chinese Academy of Agricultural SciencesAnyang, China; ^4^The State Key Laboratory of Protein and Plant Gene Research, College of Life Sciences, Peking UniversityBeijing, China; ^5^National Key Lab of Crop Genetic Improvement, National Center of Crop Molecular Breeding Technology, National Center of Oil Crop Improvement, College of Plant Science and Technology, Huazhong Agricultural UniversityWuhan, China

**Keywords:** cotton, PIN-formed, auxin efflux carriers, fiber development, expression patterns

## Abstract

The PIN-FORMED (PIN) protein, the most important polar auxin transporter, plays a critical role in the distribution of auxin and controls multiple biological processes. However, characterizations and functions of this gene family have not been identified in cotton. Here, we identified the *PIN* family in *Gossypium hirsutum*, *Gossypium arboreum*, and *Gossypium raimondii*. This gene family was divided into seven subgroups. A chromosomal distribution analysis showed that *GhPIN* genes were evenly distributed in eight chromosomes and that the whole genome and dispersed duplications were the main duplication events for *GhPIN* expansion. qRT-PCR analysis showed a tissue-specific expression pattern for *GhPIN*. Likely due to the *cis*-element variations in their promoters, transcripts of *PIN6* and *PIN8* genes from the At (tetraploid genome orginated from *G. arboreum*) subgenome and *PIN1a* from the Dt (tetraploid genome orginated from *G. raimondii*) subgenome in *G. hirsutum* was significantly increased compared to the transcripts in the diploids. The differential regulation of these *PIN* genes after the polyploidization may be conducive to fiber initiation and elongation. Exogenously applied auxin polar transport inhibitor significantly suppressed fiber growth, which is consistent with the essential function of these *PIN* genes for regulating cotton fiber development. Furthermore, the overexpression of *GhPIN1a_Dt*, *GhPIN6_At*, and *GhPIN8_At* in *Arabidopsis* promoted the density and length of trichomes in leaves.

## Introduction

The plant phytohormone, auxin, plays a crucial role in various developmental processes, such as root development, apical dominance, embryo formation, vascular differentiation, tropism, and plant response to internal and external stimuli (Dubrovsky et al., 2008; Mashiguchi et al., 2011; Rakusova et al., 2015; Wang H.Z. et al., 2015; Xi et al., 2016). Auxin is primarily synthesized in the apical meristems via several different pathways and then transported to other tissues via the plant vascular system (Zhao, 2010; Remy et al., 2013; Chen et al., 2014). The asymmetric distribution of auxin, which is largely dependent on an active process called directional polar auxin transport (PAT) mediated by influx and efflux carriers, has been confirmed to play a vital role in controlling plant growth and development (Friml, 2003; Wang et al., 2008; Band et al., 2014; D’Alessandro et al., 2015; Yang et al., 2015). The influx and efflux carriers of the auxin transporter that are distributed in the plasma membrane are grouped into three major categories: auxin resistant 1/like aux1 (AUX1/LAX) influx carriers, P-glycoprotein (MDR/PGP/ABCB) efflux/conditional transporters, and plant specific PIN-FORMED (PIN) efflux carriers. The PINs are known as the most important auxin carriers that control the auxin PAT process (Péret et al., 2012; Adamowski and Friml, 2015; Yue et al., 2015; Row et al., 2016). Recently, PIN-LIKES (PILS) family, which are localized to the endoplasmic reticulum and involved in the regulation of intracellular auxin homeostasis, was characterized as a novel putative auxin carriers (Barbez et al., 2012; Feraru et al., 2012).

In *Arabidopsis*, the *PIN* gene family, including eight members, is divided into two types: the PIN proteins with long hydrophilic loop (long PIN proteins) and with short hydrophilic loop (short PIN proteins). Both PIN types have been reported to participate in various developmental processes (Petrásek et al., 2006; Habets and Offringa, 2014; Wang H.Z. et al., 2015; Omelyanchuk et al., 2016). The long PIN proteins (AtPIN1–4 and AtPIN7) are situated in the plasma membrane and participate in root development, apical shoot establishment, and various tropic responses (Friml et al., 2002; Robert et al., 2013). AtPIN5 and AtPIN8, are localized in the endoplasmic reticulum and are involved in the homeostasis of intracellular auxin (Mravec et al., 2009; Ding et al., 2012). Interestingly, AtPIN6 is found to localize in both the plasma membrane and endoplasmic reticulum (Simon et al., 2016). Moreover, the *PIN* genes have been reported to be involved in the hormone signaling and abiotic stress responses, such as drought, salt and dehydration (Willige et al., 2011; Guo et al., 2015; Li F. et al., 2015).

The *PIN* gene family has been extensively studied using phylogenetic analyses and expression profiles in monocots and eudicots. In rice, 12 *PIN* genes have been identified, including four *PIN1* and one *PIN2*, which belong to the long PINs, and three *PIN5* and one *PIN8*, which belong to the short PINs and three monocot-specific PINs (*PIN9, PIN10a, PIN10b*) (Wang et al., 2009; Lu et al., 2015). Until now, 10 *PINs* in potato, 23 *PINs* in soybean, and 12 *PINs* in maize have been identified, and their expressions exhibit a tissue-specific pattern (Yue et al., 2015). *ZmPIN1d* is predominantly expressed in the shoot apical meristem and inflorescence meristem during the flowering stage, whereas the *ZmPIN9* transcript was primarily detected in the root endodermis and pericycle (Forestan et al., 2012). *GmPIN9*, a legume-specific *PIN* gene, may contribute to auxin redistribution in the soybean root under various abiotic stresses (Wang Y.Q. et al., 2015). *StPINs* play a major role in the swelling stolon during tuber development (Roumeliotis et al., 2013). In *Sorghum bicolor* (a monocot), 11 *PIN* genes have been characterized, including at least three *PIN1* and three *PIN5* genes, suggesting that not only monocot-specific PINs but also long PINs and short PINs exist together in monocots (Shen et al., 2010).

Cotton is one of the most important industrial crops in the world and its fibers are the main resource for the textile industry, which contributes more than 10 billion dollars annually to the global economy. Despite the extreme importance of PINs, the origins, characters and functions of these genes in cotton are still largely unknown. Taking advantage of the whole genome sequence of three cotton species that were recently completed (Paterson et al., 2012; Wang K. et al., 2012; Li et al., 2014; Li K. et al., 2015; Zhang T.Z. et al., 2015), we analyzed the *PIN* gene family comprehensively, including the phylogenic relationship, chromosomal distribution and gene duplication of the *PIN* genes. Furthermore, we suggest that *GhPIN1a_Dt*, *GhPIN6_At*, and *GhPIN8_At* may have played an important role in the fiber growth during the evolution and domestication of cotton. This is because higher transcriptional levels have only been observed in longer fiber cells in allotetraploid cotton *Gossypium hirsutum*, rather than in its diploid ancestors, *Gossypium arboreum* and *Gossypium raimondii*, which might result from the differential regulation of *GhPIN1a_Dt*, *GhPIN6_At*, and *GhPIN8_At*. The overexpression of *GhPIN1a_Dt*, *GhPIN6_At*, and *GhPIN8_At* in *Arabidopsis* enhanced the density and lengths of trichomes in leaves. Our study provides a basis to systematically elucidate the evolution and function of *PINs* in cotton and to effectively characterize the precise biological roles of *PINs* and their utilization in future cotton breeding programs.

## Materials and Methods

### Plant Materials and Growth Condition

*Gossypium hirsutum* (Xuzhou 142), *G. arboreum* (Shixiya 1), and *G. raimondii* (CMD10) that were used in this study were grown in a climate-controlled greenhouse with a 16 h light and 8 h dark cycle at 30°C, as previously reported (Shi et al., 2006). For tissue-specific expression patterns, the roots, stems, leaves, and flowers that were collected from the same growth stage of *G. hirsutum* and *G. arboreum* were immediately frozen and stored in liquid nitrogen after harvest.

### Sequence Retrieval, Multiple Sequence Alignment, and Phylogenetic Analysis

The cotton genome sequences were downloaded from the CottonGen website^1^. The *Arabidopsis* genome sequence was acquired from TAIR 10^2^. Other plant genome sequences that were used in this study were retrieved from Phytozome^3^. We used the HMMER software with default parameters and the conserved PIN transmembrane domain (PF03547.13) to search for corresponding protein sequences. The PIN sequences were further identified based on a homology search using the BLAST program. Multiple sequence alignments of all identified PINs in this study were performed using ClustalX (Thompson et al., 1997). A phylogenetic tree of deduced amino acid sequences was constructed using the neighbor-joining algorithm with default parameters and 1000 bootstrap replicates in MEGA 5.0^4^. The transmembrane topologies of the PINs were predicted using the TMHMM web server with corresponding parameters^5^.

### Gene Structure and Chromosoman Mapping

For the exon–intron structural analysis of the *PIN* genes, the coding sequences were compared with their genomic DNA sequences and the structure diagrams were generated using the GSDS server^6^. The conserved domains were predicted using the Simple Modular Architecture Research Tool (SMART^7^) and Pfam^8^ (Hu et al., 2015). The chromosome positions of all *PINs* in *G. hirsutum* were retrieved from gene annotation files that were downloaded from the CottonGen website. The map of the *PIN* gene distribution along the chromosomes was drawn from the top to the bottom using the CIRCOS program (Zou et al., 2013). We used MCScanX software for the detection and evolutionary analysis of *PIN* gene synteny and collinearity (Wang Y. et al., 2012).

### RNA Extraction and Quantitative RT-PCR (qRT-PCR) Analysis

The plant materials that were harvested as described above were frozen in liquid nitrogen and then ground to a fine powder with a mortar and pestle using a modified method (Jin et al., 2013; Liu et al., 2015). The total RNA was extracted using the PureLink^TM^ RNA mini kit (Invitrogen, Lot no.1687455) according to the manufacturer’s instructions, and the cDNA was reverse-transcribed from 5 μg of the total RNA as previously reported (Ji et al., 2003). In the qRT-PCR experiments, each gene was run in three biological and three technical replicates with the following reaction parameters: 95°C for 10 min, followed by 40 cycles of 95°C for 10 s and 56°C for 30 s. A melting curve was generated from 65 to 95°C. SigmaStat software was used for one-way statistical variance analysis. The *UBQ7* cotton gene was employed as the internal control for all related results.

### *In vitro* Ovule Culture

The *in vitro* ovule culture was performed as previously reported (Xiao et al., 2016). *N*-1-naphthylphtha-lamic acid (NPA, Sigma) was added to the culture medium with different concentrations as in the previous experimental design. Fiber lengths from cultured ovules were manually measured under a bright field microscope.

### Vector Construction and Plant Transformation

The full length (from the start codon to the stop codon) of each of the *GhPIN1a_Dt*, *GhPIN6_At*, and *GhPIN8_At* genes was amplified from the cDNA using the following gene-specific primers, respectively: 5′ primer: ACGGGGGACTCTAGAGGAT CCATGATCACTTTAACAGATTTTTACC 3′ primer: TCATA TACCCAATAAAATGTAGTACAGCTCTGTCGACTGTGGTA CCTTA 5′ primer: ACGGGGGACTCTAGAGGATCCATGATA ACAGGGGGTGATTTTTACA 3′ primer: GTATTACATACTTT TAGGCATATGAAGCTCTGTCGACTGTGGTACCTTA 5′ primer: ACGGGGGACTCTAGAGGATCCATGATTTCCCTGG CAGATGTTTATC 3′ primer: TCACAATGCTAAAAGAAGATA GTAGAGCTCTGTCGACTGTGGTACCTTA.

The pQG110 vector was digested using BamHI and KpnI. The product of each gene was then cloned into the digested pQG110 vector driven by the constitutive cauliflower mosaic virus 35S promoter via recombinase vazyme according to the manual, and the construct was further transformed into wild-type *Arabidopsis* plants. Transgenic plants were generated via the floral dip method and were selected on solid half-strength MS medium plates containing 50 mg/mL of the appropriate antibiotics (Zhang Y.Z. et al., 2015). The trichomes in the transgenic plant leaves were examined under a dissecting microscope (Leica MZ APO).

### Southern Blots Assay

Southern blots were performed as described previously (Jin et al., 2013). 15 μg genomic DNA was digested thoroughly using BamHI or EcoRI. After purification, the digested DNA was loaded onto electrophoretic gels for 4 h, then blotted prior to hybridization and detected using the DIG High Prime DNA Labeling and Detection Starter Kit II (Roche) with *GhPIN* gene sequences as probe sequences.

## Results

### Genome-Wide Identification of PIN Proteins in Three Different Cotton Species

Two diploid cottons, which were designated as AA genome and DD genome, diverged from a common ancestor approximately 2 to 13 million years ago (MYA) and developed genomes that differ approximately twofold in size. Approximately 1 to 2 MYA, these two genomes reunited in a common nucleus through allopolyploidization (Supplementary Figure S1), which led to the progenitor of the modern polyploidy cotton, *G. hirsutum*. This is the world’s primary commercial cotton species. Thus, allotetraploid cotton *G. hirsutum* contains two ancestral genomes, named the At and Dt subgenome. To identify all of the PIN proteins in *G. hirsutum* (AADD genome) and its two diploid ancestors, *G. arboreum* (AA genome) and *G. raimondii* (DD genome), we used the *Arabidopsis* PIN protein sequences to query the three reference genomes to screen out candidate PIN-like proteins in cotton. The collected PIN-like candidates were subjected to a further selection process based on their conserved domain using SMART. The PINs without the auxin efflux carrier domain were discarded. After this strict two-steps selection process, 17 deduced PINs were identified in *G. hirsutum*, along with 12 in *G. arboreum* and 10 in *G. raimondii*. Among the 17 PIN proteins identified in the *G. hirsutum* genome, seven originated from the At subgenome, nine from the Dt subgenome, and one was located on the scaffold (Supplementary Table S1). The lengths of the identified GhPIN proteins ranged from 127 (GhPIN8a_At) to 678 (GhPIN2_Dt) amino acids (aa) with an average length of 511 aa. The GaPIN proteins ranged from 124 (GaPIN3b) to 681 (GaPIN3) aa with an average length of 495 aa, and the GrPIN proteins ranged from 355 (GrPIN5) to 648 (GrPIN3) aa with an average length of 539 aa. We also analyzed orthologous *PIN* gene pairs in *G. hirsutum* and their corresponding diploid ancestors. Five genes from the A genome and two from the D genome were lost in *G. hirsutum*, whereas the orthologous genes of *GhPIN8a_At* and *GhPIN8a_Dt* in *G. hirsutum* were lost in its diploids, *G. arboreum* and *G. raimondii*, respectively.

### Analysis of Gene Loss and Duplication of *PIN* Genes in Cotton

**FIGURE 1:**
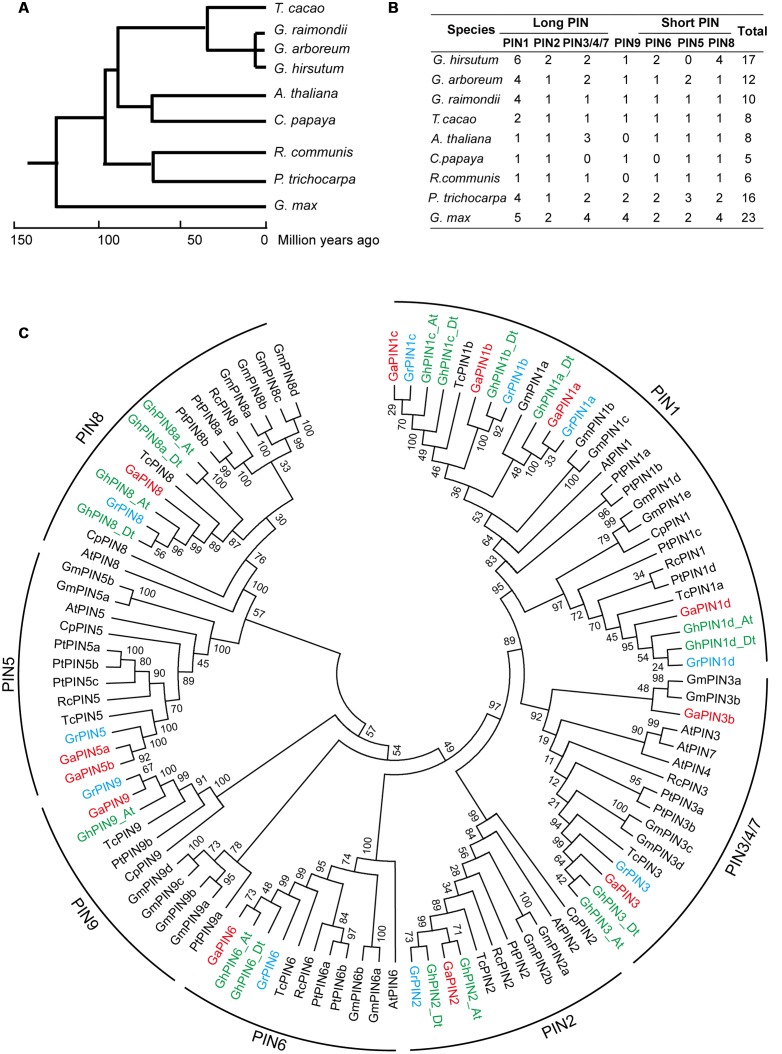
**Phylogenetic analysis of PIN auxin transporters of nine representative plant species.** **(A)** Phylogenetic analysis of nine representative species. **(B)** Number of PIN genes in the nine plant species. **(C)** Phylogenetic relationships of the PINs. The phylogenetic tree was constructed in MEGA 5.0 software using the neighbor-joining (NJ) method. The seven subfamilies of the PINs are represented by different colors. Numbers on the branches are bootstrap proportions of 1000 replicates. Ga, *Gossypium arboretum*; Gr, *Gossypium raimondii*; Gh, *Gossypium hirsutum;* Pt, *Populus trichocarpa*; Cp, *Carica papaya*; At, *Arabidopsis thaliana*; Rc, *Ricinus Communis*; Tc, *Theobroma cacao*; Gm, *Glycine max*.

To investigate the evolutionary relationships of PINs among different plant species, nine representative genome-sequenced dicotyledons that exhibited close evolutionary relationships to the three cotton species were used to construct a phylogenetic tree (**Figure 1A**). The evolutionary analysis showed that the *PIN* family genes in cotton are divided into long PINs (PIN1, PIN2, PIN3/4/7), short PINs (PIN5, PIN6, PIN8) and PIN9 (**Figure 1B**). According to the phylogenetic tree, the PIN family proteins can be divided into seven subgroups (**Figure 1C**). The *PIN1* subgroup was most prominent in cotton. In contrast to most of the plant species analyzed, the *PIN1* subgroup was extensively expanded in cotton, *Populus trichocarpa* and *Glycine max*. The *PIN8* subfamily, contributing to auxin homeosatstic and facilitating the male gametophyte development in *Arabidopsis* (Ding et al., 2012), was extensively expanded in *G. hirsutum*, which may correspond to its better agronomic. *PIN5* subgroup gene, which act antagonistically with *PIN8*, were missing in allotetraploid cotton, suggesting the loss of the gene occurred during the evolution of the *G. hirsutum* after polyploidization.

Compared with *Arabidopsis*, eight other plant species contained an additional *PIN9* subgroup, which suggests a loss of this *PIN* in *Arabidopsis* during the evolutional course. Both *PIN4* and *PIN7* subgroups were only found in *Arabidopsis*, but none were found in the other species studied, indicating that these genes may have been acquired in *Arabidopsis* after it diverged from the common ancestor of the nine plant species used for analysis in the **Figure 1A**.

**FIGURE 2:**
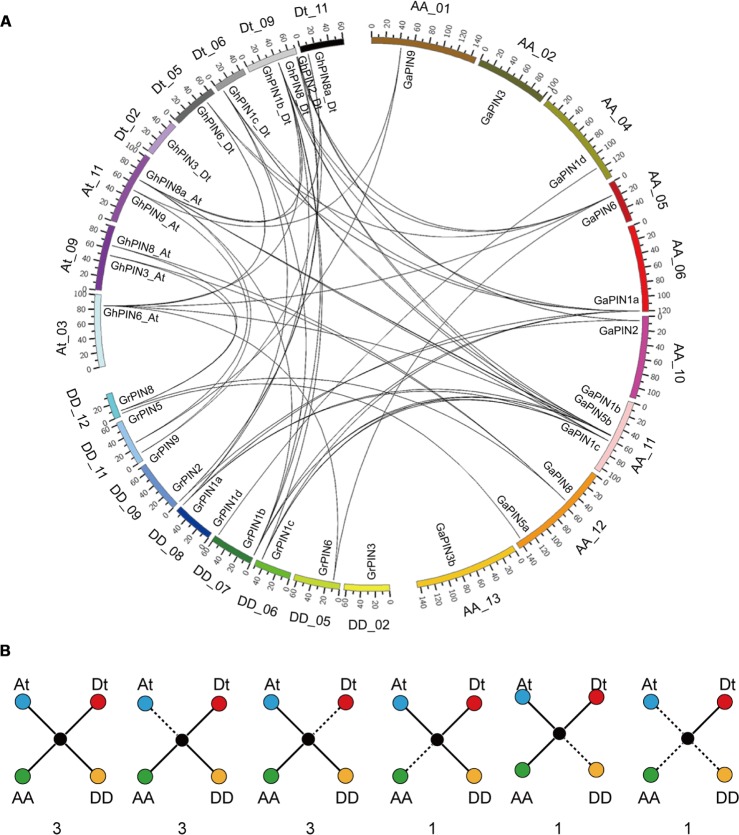
**Chromosomal distribution and evolutionary analysis of the *PIN* genes.** **(A)** Chromosomal location and analysis of *PIN* gene duplication. The CIRCOS genome visualization tool was used. Each chromosome number is provided next to the corresponding chromosome. The black lines represent duplicated gene pairs. **(B)** Scenarios and statistics of gene conservation of the three different genomes. The solid lines indicate observed genes, and the dotted lines indicate lost genes. The number of gene pairs found in the three different genomes that fit the specific model is provided below each graphic.

With the A-genome *G. arboreum*, D-genome *G. raimondii*, and the allotetraploid genome sequenced, we further explored the evolutionary history of the cotton *PIN* genes involved in gene duplication and loss. Our results showed that 12 *GhPINs* were evenly distributed on eight chromosomes, three from the At subgenome, and five from the Dt subgenome (**Figure 2A**). According to a whole genome analysis of gene duplications, we found that the whole genome duplication (WGD) and dispersed duplication, rather than the proximal duplication and tandem duplication, were the main driving forces of the *GhPIN* gene expansion (Supplementary Table S2). Specifically, nine *GhPINs* were produced via WGD, and three genes were produced via dispersed duplication among the analyzed genes.

Then, we analyzed the *PIN* gene conservation and loss based on the best match and the syntenic gene finder in MCScan. Three *PIN*s were ultra-conserved homologous gene pairs in *G. hirsutum*. Of the orthologous gene pairs, six were lost from either the At or the Dt subgenome of *G. hirsutum* and two were lost from either *G. arboreum or G. raimondii* (**Figure 2A**). This indicated that the *GhPIN* genes experienced a higher frequency of genic sequence losses and only retained either the At or Dt homologous genes during the formation of *G. hirsutum*. Compared with one gene lost in the A genome and one lost in the D genome (**Figure 2B**), the rate of gene losses was higher in the allotetraploid cotton than in both diploid species. Additionally, one gene was only present in the Dt subgenome (**Figure 2B**).

### Gene Structure and Transmembrane Topology among PIN Proteins

**FIGURE 3:**
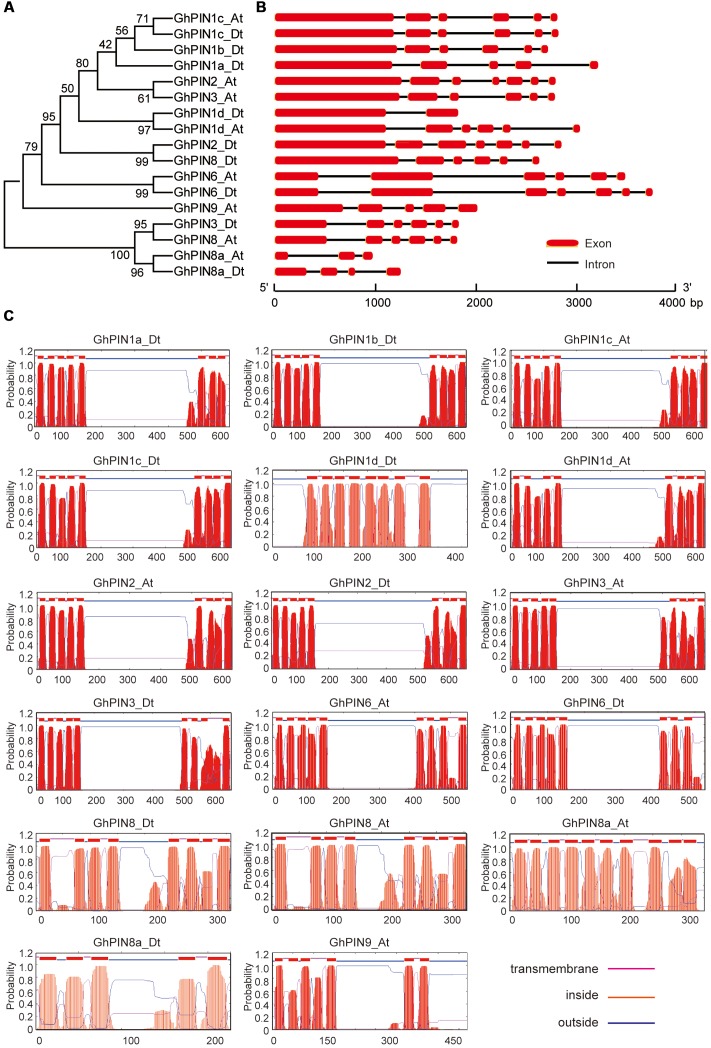
**Phylogenetic relationships, gene structures, and transmembrane topology prediction for the GhPINs.** **(A)** Phylogeny of 17 PIN proteins in *Gossypium hirsutum*. The phylogenetic tree (left panel) was constructed in MEGA 5.0 using the neighbor-joining (NJ) method with 1000 bootstrap replicates. **(B)** The gene structures (right panel) were drawn with the Gene Structure Display Server 2.0^42^. The exons and introns are marked with red boxes and black lines, respectively. The scale bar is shown at the bottom. **(C)** Transmembrane topology prediction of the GhPIN proteins. The transmembrane helices of the GhPIN proteins were predicted using the TMHMM Server v.2.0 (http://www.cbs.dtu.dk/services/TMHMM/). The red peaks indicate the predicted transmembrane helices.

To better understand the similarity and diversity of the gene structures and transmembrane topology of GhPINs, we constructed a phylogenetic tree with the deduced amino acids of GhPINs (**Figure 3A**) and investigated the exon/intron structures of *GhPIN* genes. In general, *GhPINs* possessed at least one intron and the structural patterns were highly conserved between long PINs and short PINs (**Figure 3B**). Although *GhPIN6_At* and *GhPIN6_Dt* genes belong to short PINs, both had five introns, including the two largest introns, in their open reading frame. The predicted transmembrane topology showed that GhPIN proteins, except for GhPIN1d_Dt, had the typical structure with two highly conserved hydrophobicity segments at the N- and C- terminus and a central hydrophilic loop within the two termini (**Figure 3C**). All GhPIN proteins possessed 8–10 transmembrane segments, except GhPIN8a_Dt, which presented six transmembrane helices. Notably, GhPIN8a_At possessed identical transmembrane segments as the other GhPINs, but these segments were evenly distributed across the entire protein, which resulted in the loss of the central hydrophilic loop.

### Tissue-Specific Expression Profiles of Cotton *PIN* Genes

**FIGURE 4:**
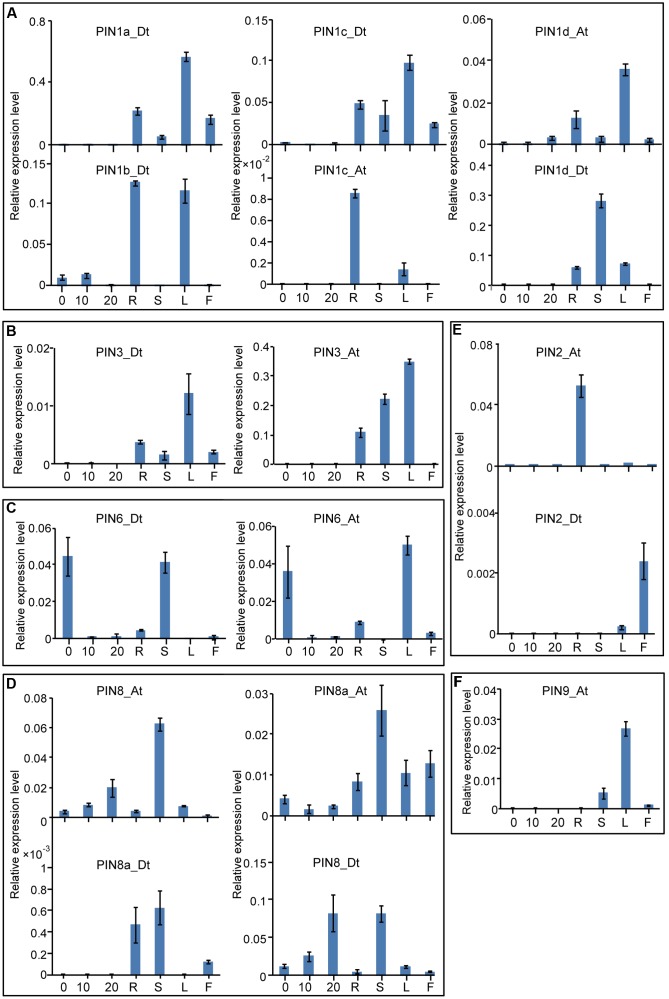
**Tissue-specific expression profiles of the *GhPIN* genes.** **(A)** Three *GhPIN* genes were expressed significantly higher in the fibers. **(B)** Three *GhPIN* genes were expressed significantly higher in the stem. **(C)** Six *GhPIN* genes were expressed significantly higher in the leaf. **(D)** Four *GhPIN* genes were expressed significantly higher in the root. **(E)** *PIN2a-At* was expressed significantly higher in the flower. The qRT-PCR analyses were performed in three biological replicates, and the error bars represent their mean values ± SE. The expression levels are given relative to cotton *UBQ7*. **(F)** *PIN9-At* was expressed significantly higher in the leaf. R, root; S, stem; L, leaf; F, flower. The primers for the qRT-PCR analyses are listed in Supplementary Table S3.

Systematic analyses of gene expression patterns can help us understand the possible physiological functions of the cotton genes. First, we investigated the expression patterns of the *GhPIN* genes in different tissues, including the root, stem, leaf, flower, and fibers at various developmental stages. As shown in **Figure 4**, *GhPIN* transcripts were widely detected and exhibit a tissue-specific expression pattern in cotton. Genes from the PIN1, PIN2, PIN3, and PIN9 subgroups were predominantly expressed in vegetative tissues, but they were barely detected in the fibers, indicating that these genes may play specific roles in regulating the development of vegetative tissues. The transcripts from the PIN6 and PIN8 subgroup genes were abundant in diversified tissues. *PIN1c_At* from the PIN1 subgroup showed a root-specific expression, whereas the other four genes from the PIN1 subgroup were notably more highly expressed in the leaf (**Figure 4A**). Two PIN3 subgroup genes, *PIN3_Dt* and *PIN3_At*, were expressed in a leaf-preferential manner (**Figure 4B**). *PIN6_Dt* and *PIN6_At*, two genes from the PIN6 subgroup, were highly expressed in the 0 day post-anthesis (DPA) ovule (**Figure 4C**). Four PIN8 subgroup genes were significantly expressed in the stem (**Figure 4D**). The transcripts of *PIN2_At* genes were significantly higher in the root, whereas *PIN2_Dt* was specifically expressed in the flower (**Figure 4E**). *PIN9_At* was specifically expressed in the leaf.

### Analysis of the Link between *PIN* and Fiber Elongation

**FIGURE 5:**
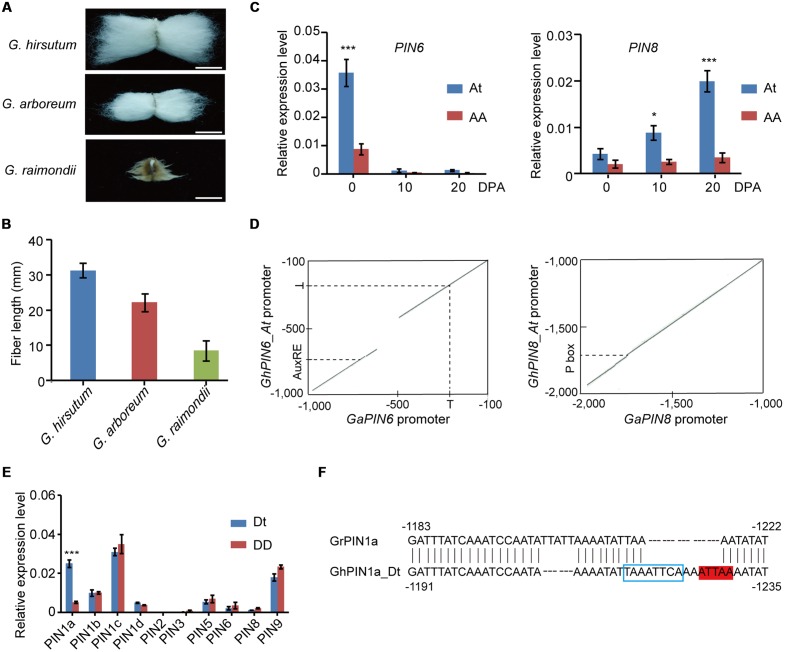
**The emergence of differential regulation of *PIN* expression in the fiber indicates its important role in fiber elongation during the evolution of cotton.** **(A)** Comparisons of mature fibers of *G. hirsutum*, *G. arboreum*, and *G. raimondii*. Bar = 1.5 cm. **(B)** Final statistics of mature fibers of *G. hirsutum*, *G. arboreum*, and *G. raimondii*. Each data is the mean of three independent experiments with a total of 10 measured samples. The error bars indicate the mean values ± SE. **(C)** Quantitative RT-PCR analysis of the *GaPIN6* and *GaPIN8* genes. ^∗^*P* < 0.05. **(D)** Dot plots showing consensus in the promoter regions of Ga*PIN6* (left) and Ga*PIN8* (right) and their orthologs from the At subgenome. T, putative TATA box; AuxRE, putative auxin response element; P box, putative pyrimidine box. **(E)** Quantitative RT-PCR analysis of the *PIN* genes using the Dt (indicated by blue lines) or D-originated (red lines) copies at the 10 DPA as the template. Three biological replicates were analyzed via qRT-PCR in **(C,E)**, and the error bars represent the mean values ± SE. The expression levels are relative to cotton *UBQ7*. DPA, day after anthesis. ^∗∗∗^*P* < 0.001. **(F)** Promoter sequence analysis of Gr*PIN1a* and its orthologs from the Dt subgenome. The blue box marks the putative HD-ZIP binding site, and the red color represents the putative ZF-HD binding site.

Fiber length is one of the key agronomic traits that determine cotton yield and quality. First, we analyzed the fiber length in *G. hirsutum* and its diploid ancestors and found pronounced differences in *G. hirsutum*, *G. arboreum*, and *G. raimondii* (**Figure 5A**). The fiber length of *G. hirsutum* was more than 3 cm, whereas *G. arboreum* and *G. raimondii* showed a substantial reduction in fiber length (**Figure 5B**). To reveal the potential clues regarding whether the differential expression of *PIN* genes, which were generated during the origin and evolution of cotton, influenced the fiber development, we performed qRT-PCR to analyze the expression level of *PIN* genes during the different growth periods of the fibers from *G. hirsutum* and *G. arboreum*. Among the seven *PIN* genes analyzed, the *PIN6* and *PIN8* genes that originated from the At subgenome of *G. hirsutum*, rather than their homologs from the A genome of *G. arboreum*, were preferentially expressed in the fiber initiation and elongation stages (**Figure 5C** and Supplementary Figure S2). A comparative analysis of promoter sequences provides insights into the possible regulation mechanisms for the differential expression of *PIN6* and *PIN8* between *G. hirsutum* and *G. arboreum*. Compared with the *GaPIN6* promoter, a large DNA fragment deletion was observed in the *GhPIN6_At* promoter. Additionally, a point mutation was identified in the *GaPIN6* promoter, which resulted in the loss of an auxin response element (**Figure 5D** and Supplementary Figure S3). Although the *GhPIN8_At* promoter displayed a 98.1% similarity with the *GaPIN8* promoter, we observed the loss of a pyrimidine box in the *GaPIN8* promoter (**Figure 5D** and Supplementary Figure S4), which was reported to play an important role in regulating the expression of *Ces3–4* gene from the At subgenome (Li F. et al., 2015).

We further compared the transcripts of the other 10 *GhPIN* genes with their corresponding orthologs in *G. raimondii*. In the qRT-PCR analysis, nine *PIN* genes showed no substantial differences in their transcriptional levels between the two cotton species, and only the expression level of *PIN1a* was approximately fivefold higher than its ortholog in *G. raimondii* (**Figure 5E**). Further promoter scrutiny revealed that several short sequences were missing in the *GrPIN1a* promoter (indels of 8–16 bp) compared with the *GhPIN1a_Dt* promoter, which resulted in the loss of a putative HD-ZIP binding site and a putative ZF-HD binding site (**Figure 5F**). Our results imply that the *cis*-element variations generated in these gene promoters during evolution may be coupled with the high-level expressions of the *GhPIN6_At*, *GhPIN8_At*, and *GhPIN1a_Dt* genes in allotetraploid cotton.

Increased expressions of *PIN* genes in longer fiber cells suggest that the auxin transport may promote fiber elongation. Therefore, we needed to confirm whether *PINs* play important roles in cotton fiber development. We utilized an auxin polar transport inhibitor to block the functions of the PINs and measured the fiber lengths with an ovule culture system *in vitro*. When NPA, an inhibitor of auxin polar transport, was added to the standard ovule culture media for 13 days, we observed a significant reduction in fiber elongation (Supplementary Figure S5A). Furthermore, the fiber lengths were reduced in a dose-dependent manner and the addition of 80 μM NPA to the culture media almost entirely blocked fiber growth (Supplementary Figure S5B). However, the application of different concentrations of NPA did not affect the final size of the cultured ovules (Supplementary Figure S5C). qRT-PCR analysis showed that the transcription levels of most *PIN* genes decreased after the application of NPA (Supplementary Figure S5D). These results suggest that the PIN proteins are essential for cotton fiber cell elongation.

### The Overexpression of the *GhPIN1a_Dt*, *GhPIN6_At* and *GhPIN8_At* Genes Increase the Density and Lengths of Trichomes in Leaves

**FIGURE 6:**
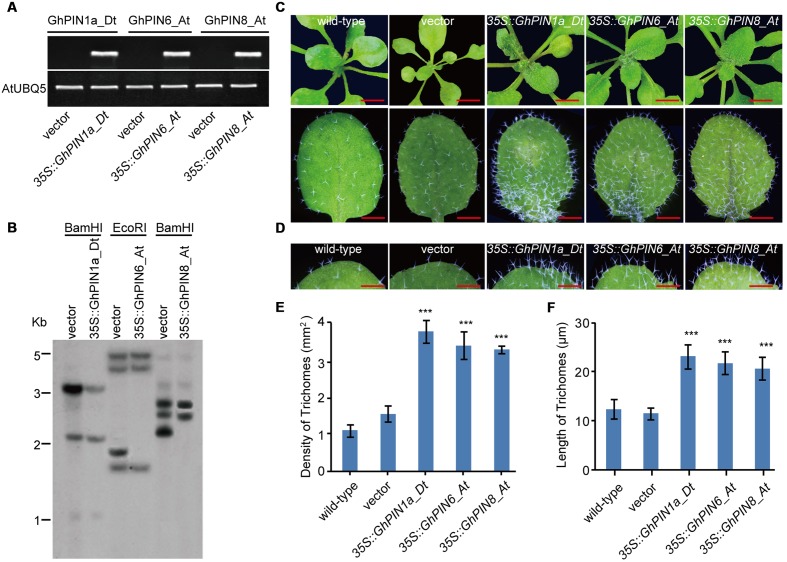
**Density and lengths of trichomes in leaves from *GhPIN1a_Dt*, *GhPIN6_At*, and *GhPIN8_At* transgenic plants.** **(A)** PCR verification of *GhPIN1a_Dt*, *GhPIN6_At*, and *GhPIN8_At* transgenic plants. **(B)** Southern blot analysis of *GhPIN1a_Dt*, *GhPIN6_At* and *GhPIN8_At* transgenic plants. **(C)** Trichome phenotypes of transgenic plants. The scale bars in the upper and lower panels are 0.5 mm and 100 μm, respectively. **(D)** Trichome lengths of the transgenic and control plants. The scale bars is 50 μm. **(E)** Comparison of the densities of trichomes in the leaves from the transgenic and control plants. Each experiment was performed in three biological replicates, and the error bars represent the mean ± SE. ^∗∗∗^*P* < 0.001. **(F)** Comparison of the lengths of trichomes in the leaves from the transgenic and control plants. Each experiment was performed in three biological replicates, and the error bars represent the mean ± SE. ^∗∗∗^*P* < 0.001. Vector, the construct without *PIN* gene as the control.

To further investigate the functions of the *GhPIN1a_Dt*, *GhPIN6_At*, and *GhPIN8_At* genes identified through their differential expression patterns of the three cotton species, we generated three transgenic lines with *GhPIN1a_Dt*, *GhPIN6_At* or *GhPIN8_At* overexpressed in *Arabidopsis* that was driven by the constitutive 35S promoter (**Figure 6A**), and the expression level of *GhPIN1a_Dt*, *GhPIN6_At* and *GhPIN8_At* was 123, 48, and 87% of the *Arabidopsis UBQ5* gene, respectively (Supplementary Figure S6). Meanwhile, the three transgenic lines used for phenotypic analysis were with single copy verified by southern blotting experiment (**Figure 6B**). In contrast to the transgenic lines carrying the empty vector, the three transgenic plants that carried the *35S::GhPIN1a_Dt*, *35S::GhPIN6_At or 35S::GhPIN8_At* constructs showed a considerable increase of number and length of trichomes, an organ similar to cotton fiber, in the leaves (**Figures 6C,D**). Furthermore, the calculated density of trichomes per leaf in the *35S::GhPIN1a_Dt*, *35S::GhPIN6_At* or *35S::GhPIN8_At* transgenic lines increased to 250, 220, or 210% of the level in the leaf of transgenic line carrying the empty vector, respectively (**Figure 6E**). Compared with the transgenic line carrying the empty vector, the *35S::GhPIN1a_Dt*, *35S::GhPIN6_At* or *35S::GhPIN8_At* displayed longer trichomes in the leaves (**Figure 6F**). These results suggest that the *GhPIN1a_Dt*, *GhPIN6_At* and *GhPIN8_At* genes possess biological activity for cell elongation *in vivo*, which strongly suggests that the high expression of the three *PINs* in *G. hirsutum* contributes to cotton fiber development.

## Discussion

For the first time, we simultaneously identified the *PIN* genes in three representative types of cotton, allotetraploid cotton *G. hirsutum* and its diploid ancestors, *G. arboreum* and *G. raimondii*, which provides significant insight into the evolution and functions of in *PINs* in cotton. This study showed that the *PIN1* and *PIN8* subgroup genes in cotton were significantly expanded (**Figure 1B**), indicating the functional innovation of these *PIN* subgroup genes in cotton. Conversely, the *PIN5* subgroup genes were only lost in *G. hirsutum* but were found in *G. arboreum* and *G. raimondii* and in other plant species, showing a close evolutionary relationship to *Gosspium*. This result implies that the *PIN5* subgroup genes were present in the common ancestor of these plant species and even in the most recently common ancestors of cotton (ancestral *Gossypium*) before the divergence of the diploid cottons (**Figure 1C**). After the polyploidization of the two ancestral diploid cotton types, the *PIN5* subgroup gene was gradually lost during the evolution or domestication history of allotetraploid cotton, indicating that the *PIN5* gene might be not essential for *G. hirsutum* development. Although two whole genomic duplication events occurred in cotton, many *PIN* subgroups contain a similar amount of genes compared with other plant species that have a close evolutional relationship.

The chromosomal distribution of the *PIN* genes showed that 12 of the 17 genes were evenly located on eight chromosomes (**Figure 2A**). Among the 12 analyzed genes, nine *PINs* were produced by WGD and three by dispersed duplication (Supplementary Table S3). This implies that the two types of duplication events are the main contributors of the *PIN* gene expansion in cotton. More *PIN* genes were lost in *G. hirsutum* instead of in the two diploid ancestors, suggesting that a higher rate of gene losses occurred in *G. hirsutum*. This confirms the previous notion that the allotetraploid genome experienced a higher frequency of genic sequence losses than the diploid genomes during polyploidization (Li F. et al., 2015; Zhang T.Z. et al., 2015; He et al., 2016). Therefore, *PINs* may play a different role in allotetraploid cotton than in the diploid ancestors.

The transcription profiling of cotton fibers showed that the transcriptomes of cotton fibers are extraordinarily complex, involving 1000s of genes that vary in expression level between *G. hirsutum* and its diploid ancestors, *G. arboreum* and *G. raimondii* (Hovav et al., 2008). Previous work revealed that the ethylene biosynthetic gene, 1-aminocyclopropane-1-carboxylic acid oxidase (*ACO*), and the cell wall biosynthetic gene, cellulose synthase (*Ces*), play a vital role in cotton fiber elongation (Li F. et al., 2015). A comparative analysis showed different expression patterns of these genes in the three representative cotton species, resulting in *cis*-element variations in their promoter, including the indel and nucleotide transitions, which generate new transcriptional factor binding sites. The analysis of *PINs* in cotton showed that, apart from the genes concerned with ethylene and cell wall synthesis, the elevated expression patterns of *PIN* genes also contribute to the cotton fiber elongation in *G. hirsutum* (**Figure 5**), especially, *PIN1a*, *PIN6*, and *PIN8.* These are driven by the promoter with new *cis*-elements that were acquired during evolution and domestication. These results suggest that the differential expression pattern of these genes that are related to the fiber elongation, which were derived from the force of the evolution or domestication, may be a common phenomenon and facilitate the emergence of fibers in cotton.

No fiber was found in *G. raimondii*, and the fiber length in *G. arboreum* was only 1.3–1.5 cm. However, in *G. hirsutum*, the two genomes reunited in a common nucleus via allopolyploidization, resulting in its fiber length of more than 3 cm. Our results showed that in the three functional *PINs* identified, the *G. hirsutum PIN1a* originated from the Dt subgenome, whereas *PIN6* and *PIN8* originated from the At subgenome. This suggests that allopolyploidization facilitates the acquisition of both advantages of the two diploid cotton types, which contributes to fiber initiation and elongation in *G. hirsutum*.

Auxin is known to play essential roles in enhancing cotton fiber cell initiation (Zhang et al., 2011), which also rely on the auxin transporters. On that basis, we further showed that *PIN* genes may also have a positive effect on the fiber elongation in addition to initiation. This suggests *PINs* play bifunctional roles in fiber development. Our work provides evolutionary and functional insights into the roles of *PINs* in cotton fiber development from both evolutionary and functional aspects and thus may help to improve both cotton fiber yields and the quality of future research and development efforts for improving cotton.

## Author Contributions

YZ and GX conceived all of the experiments and analyzed the data. PH, ZY, and GH performed the experiments, drafted the manuscript, and prepared the figures. GX and JY wrote and reviewed the manuscript. LW and HX analyzed the data. PZ and CP contributed to the manuscript preparation. All authors reviewed the manuscript.

## Conflict of Interest Statement

The authors declare that the research was conducted in the absence of any commercial or financial relationships that could be construed as a potential conflict of interest.

## Footnotes

1 https://www.cottongen.org

2 http://www.arabidopsis.org

3 https://phytozome.jgi.doe.gov/pz/portal.html

4 http://www.megasoftware.net

5 http://www.cbs.dtu.dk/services/TMHMM

6 http://gsds.cbi.pku.edu.cn

7 http://smart.embl-heidelberg.de/

8 http://pfam.xfam.org
