# Supplementary material for: A Genome-Scale Analysis of the PIN Gene Family Reveals Its Functions in Cotton Fiber Development
Source: Front Plant Sci. 2017 Mar 30;8:461. doi: 10.3389/fpls.2017.00461 (PMC5371604; doi:10.3389/fpls.2017.00461)
Supplement: Supplementary file 1 [file Data_Sheet_1.PDF]

Supplementary Material

A genome-scale analysis of the *PIN* gene family reveals its functions in cotton fiber development

Yuzhou Zhang[1, 2,*], Peng He[1,*], Zuoren Yang[3,*], Gai Huang[4], Limin Wang[5], Chaoyou Pang[3], Hui Xiao[1], Peng Zhao[1], Jianing Yu[1], Guanghui Xiao[1]

[1] Key Laboratory of the Ministry of Education for Medicinal Plant Resources and Natural Pharmaceutical Chemistry, National Engineering Laboratory for Resouce Development of Endangered Crude Drugs in the Northwest of China, College of Life Sciences, Shaanxi Normal University, Xi’an 710119, China

[2] Institute for Advanced Studies/College of Life Sciences, Wuhan University, Wuhan 430072, China

[3] State Key Laboratory of Cotton Biology, Cotton Research Institute, Chinese Academy of Agricultural Sciences, Anyang 455000, China

[4] The State Key Laboratory of Protein and Plant Gene Research, College of Life Sciences, Peking University, Beijing, 100871, China

[5] National Key Lab of Crop GeneticImprovement, National Center of Crop Molecular Breeding Technology, National Center of Oil Crop Improvement (Wuhan), College of Plant Science and Technology, Huazhong Agricultural University,Wuhan, 430070, China

*These authors contributed equally to this work.

Correspondence: guanghuix@snnu.edu.cn; jnyu@snnu.edu.cn.

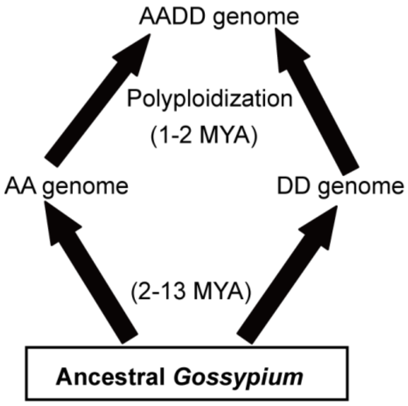

Figure S1 | Evolutionary analysis of allotetraploid cotton. The AA and DD genomes evolved ~2 to 13 million years ago (MYA) after their divergence from a common ancestor and were further elaborated into allotetraploid cotton (AADD genome) after the polyploidization ~1 to 2 MYA.

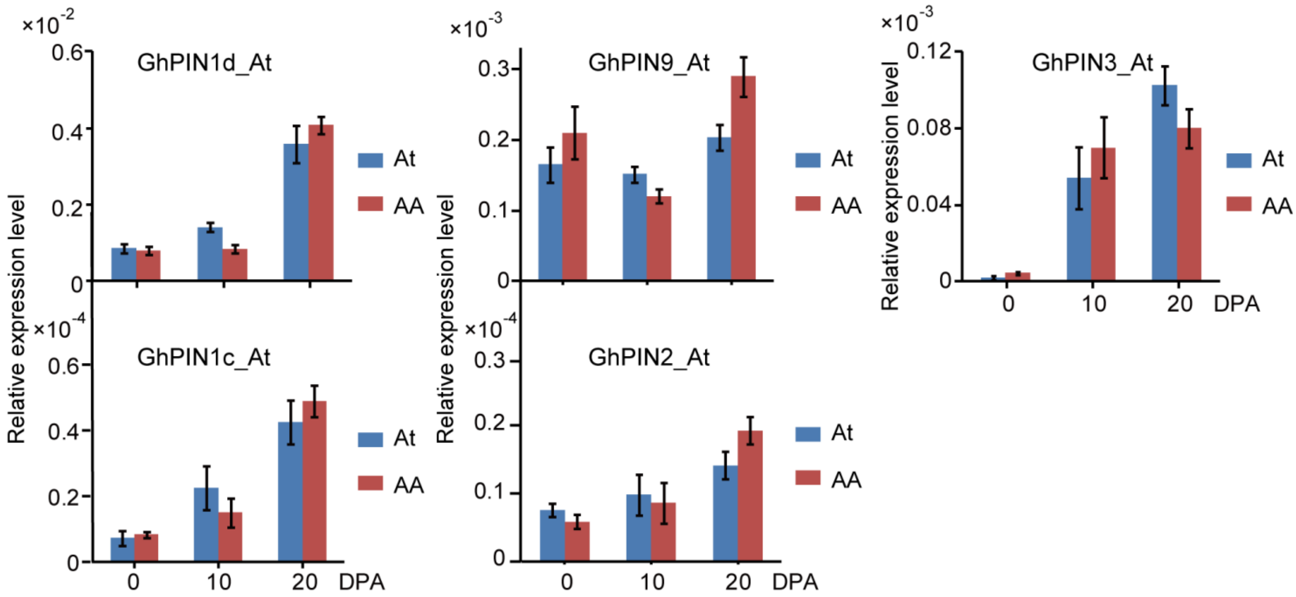

Figure S2 | Expression profiles of the other five *PIN* genes in the fibers during different developmental stages. Three biological replicates were analyzed via qRT-PCR, and the error bars represent the mean ± SE. The expression levels are relative to cotton *UBQ7*. DPA, day after anthesis.

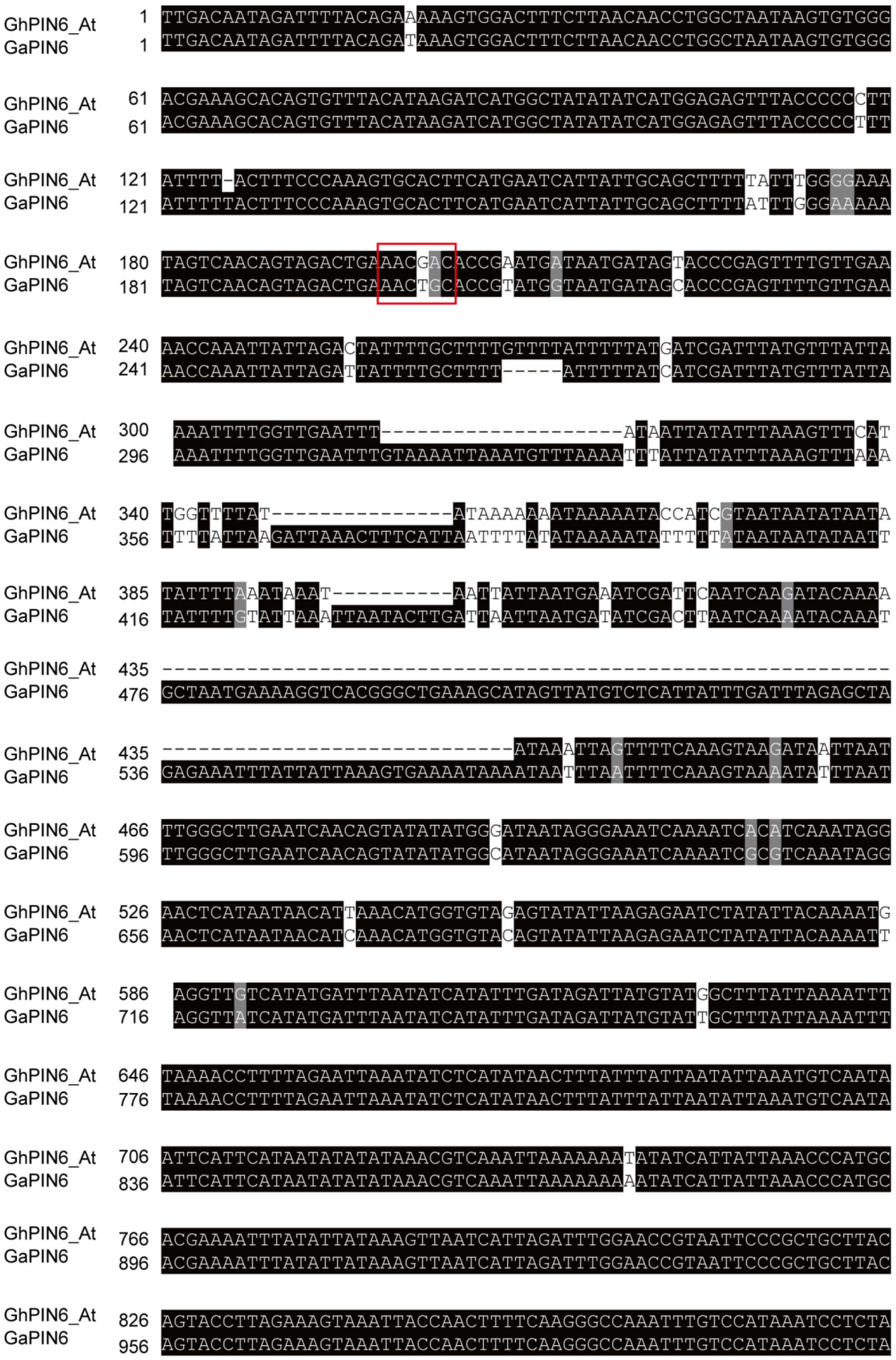

Figure S3 | Sequence alignment of the *PIN6* promoter from *G. arboreum* (Ga) and its orthologues from *G. hirsutum* (Gh). The sequence highlighted in the red

box is the auxin response element, which was predicted using PlantCARE software. The dashed lines indicate the lost sequences and indels.

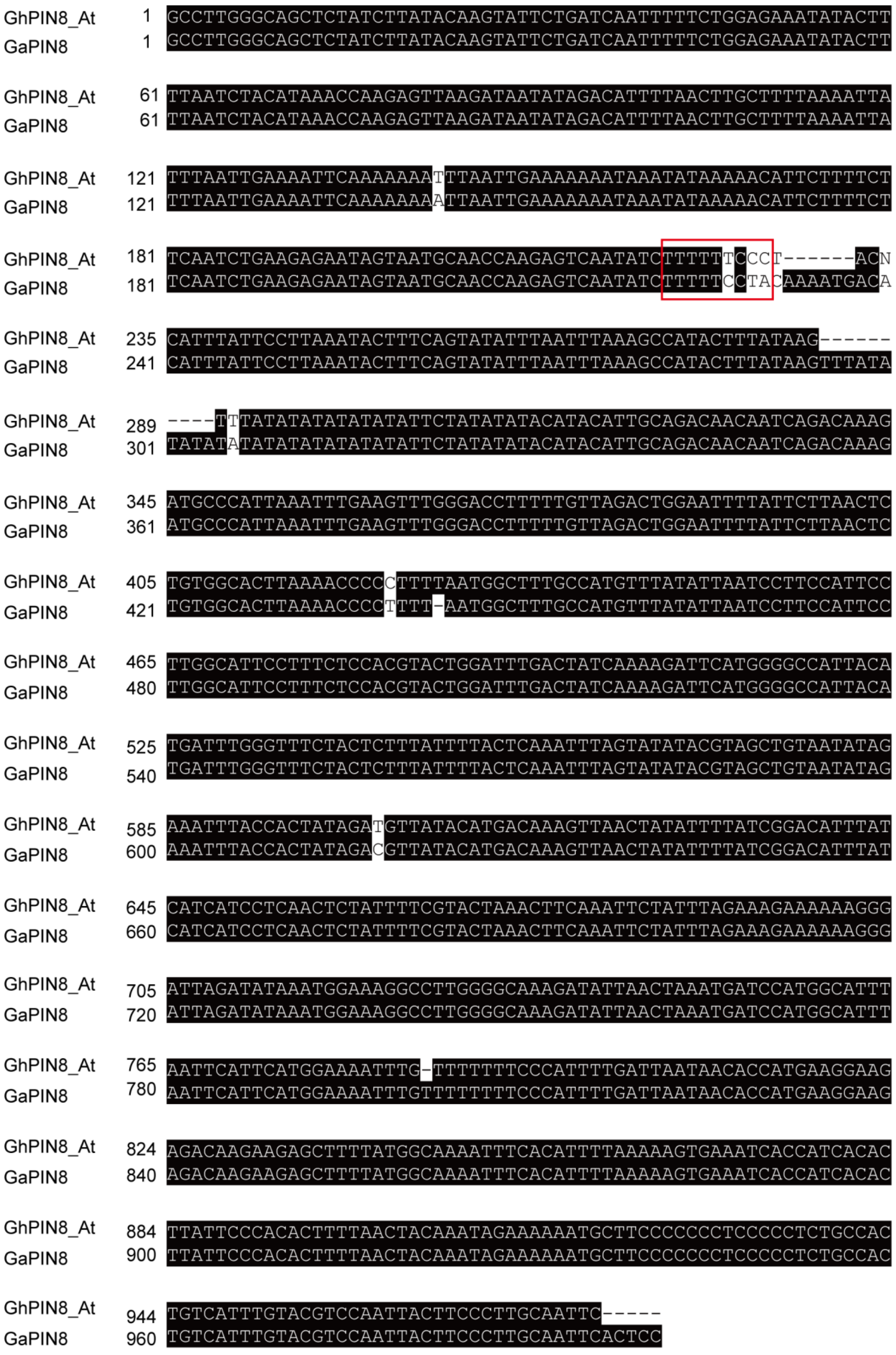

Figure S4 | Sequence alignment of the *PIN8* promoter from *G. arboreum* (Ga) and its orthologues from *G. hirsutum* (Gh). The sequence highlighted in the red

box is the pyrimidine box, which was predicted using PlantCARE software. The dashed lines indicate the lost sequences and indels.

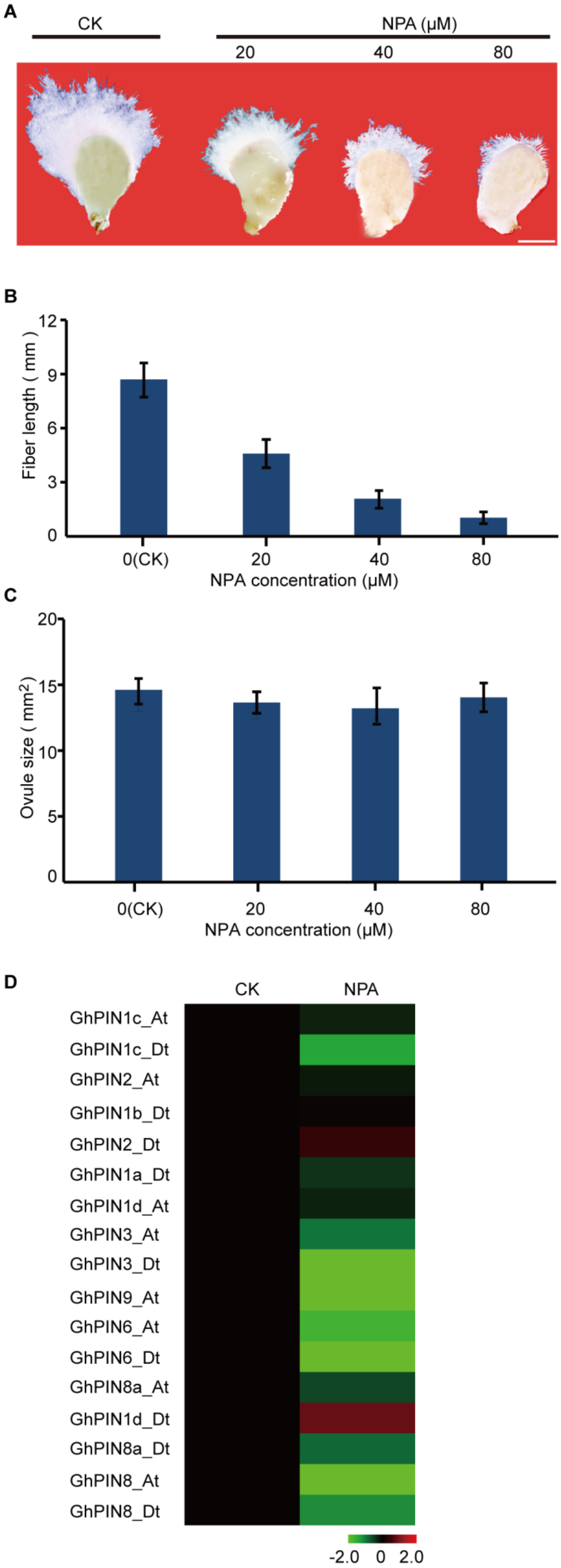
A
CK
NPA (μM)
20
40
80
B
Fiber length (mm)
12
9
6
3
0
0(CK)
20
40
80
NPA concentration (μM)
C
Ovule size (mm2)
20
15
10
5
0
0(CK)
20
40
80
NPA concentration (μM)
D
CK
NPA
GhPIN1c_At
GhPIN1c_Dt
GhPIN2_At
GhPIN1b_Dt
GhPIN2_Dt
GhPIN1a_Dt
GhPIN1d_At
GhPIN3_At
GhPIN3_Dt
GhPIN9_At
GhPIN6_At
GhPIN6_Dt
GhPIN8a_At
GhPIN1d_Dt
GhPIN8a_Dt
GhPIN8_At
GhPIN8_Dt
-2.0
0
2.0

Figure S5 | Exogenous NPA inhibits fiber cell elongation. (**A**) Phenotypes of 13-d-old wild-type ovules (collected at 1 DPA) cultured with or without (CK) NPA supplementation. Bar = 5 mm. (**B**) Final fiber lengths after NPA treatment that were measured at the end of the 13-d culture period. (**C**) Ovule sizes after NPA treatment that were measured at the end of the 6-d culture period. Each value in (**B**) and (**C**) was acquired in three biological replicates from a total of 30 fiber cells and 10 ovules that were evaluated. The error bars represent the mean ± SE. (**D**) The effect of 80 μM NPA on *GhPIN* gene expression. Relative expression levels were determined after normalizing all data to that of CK, which was set to 1.0. Levels of different colors were shown on a $\log_2$ scale.

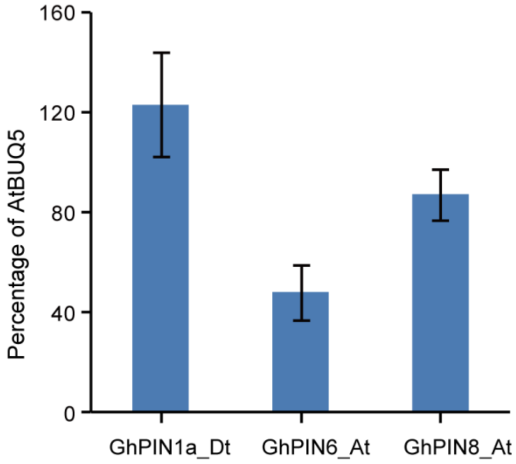

Figure S6 | Identification of *GhPIN1a_Dt*, *GhPIN6_At* and *GhPIN8_At* transgenic plants. The expression level of *GhPIN1a_Dt*, *GhPIN6_At* and *GhPIN8_At* in transgenic plants. The expression levels are given relative to *Arabidopsis UBQ5* (*AtUBQ5*) gene. The qRT-PCR analyses were performed in three biological replicates, and the error bars represent their values ± SE.

Table S1 | Analysis of *G. hirsutum PIN* gene family and its orthologs in AA and DD cotton genomes.

Gene ID	Length of amino acid	Gene name	Orthologous	Length of amino acid	Gene name
NA	NA	NA	Cotton_A_01439	604	GaPIN1a
NA	NA	NA	Cotton_A_07609	614	GaPIN1b
CotAD_70220	605	GhPIN1c_At	Cotton_A_13051	638	GaPIN1c
CotAD_04916	585	GhPIN1d_At	Cotton_A_14311	630	GaPIN1d
CotAD_43404	630	GhPIN2_At	Cotton_A_29759	675	GaPIN2
CotAD_74791	638	GhPIN3_At	Cotton_A_40424	681	GaPIN3
NA	NA	NA	Cotton_A_25858	124	GaPIN3b
NA	NA	NA	Cotton_A_16015	355	GaPIN5a
NA	NA	NA	Cotton_A_33451	332	GaPIN5b
CotAD_69056	547	GhPIN6_At	Cotton_A_19614	448	GaPIN6
CotAD_47634	355	GhPIN8_At	Cotton_A_29004	271	GaPIN8
CotAD_21235	127	GhPIN8a_At	NA	NA	NA
CotAD_58548	470	GhPIN9_At	Cotton_A_30421	412	GaPIN9
CotAD_21821	583	GhPIN1a_Dt	Gorai.008G290000.1	604	GrPIN1a
CotAD_02796	617	GhPIN1b_Dt	Gorai.007G003800.1	617	GrPIN1b
CotAD_03159	605	GhPIN1c_Dt	Gorai.006G191800.1	605	GrPIN1c
CotAD_04915	472	GhPIN1d_Dt	Gorai.007G352200.1	585	GrPIN1d
CotAD_23365	678	GhPIN2_Dt	Gorai.009G001600.1	630	GrPIN2
CotAD_68019	668	GhPIN3_Dt	Gorai.002G179000.1	648	GrPIN3
NA	NA	NA	Gorai.012G029000.1	355	GrPIN5
CotAD_05841	526	GhPIN6_Dt	Gorai.005G082500.1	547	GrPIN6
CotAD_40873	358	GhPIN8_Dt	Gorai.012G056200.1	358	GrPIN8
CotAD_15657	226	GhPIN8a_Dt	NA	NA	NA
NA	NA	NA	Gorai.011G156600.1	451	GrPIN9

Table S2 | Analysis of duplication events in *G. hirsutum PIN* genes located in chromosomes.

Gene ID	Gene name	Duplication type
CotAD_02796	GhPIN1b_Dt	Whole Genome/Segmental Duplication
CotAD_03159	GhPIN1c_Dt	Whole Genome/Segmental Duplication
CotAD_23365	GhPIN2_Dt	Whole Genome/Segmental Duplication
CotAD_69056	GhPIN6_At	Whole Genome/Segmental Duplication
CotAD_05841	GhPIN6_Dt	Whole Genome/Segmental Duplication
CotAD_47634	GhPIN8_At	Whole Genome/Segmental Duplication
CotAD_40873	GhPIN8_Dt	Whole Genome/Segmental Duplication
CotAD_15657	GhPIN8a_Dt	Whole Genome/Segmental Duplication
CotAD_21235	GhPIN8a_At	Whole Genome/Segmental Duplication
CotAD_74791	GhPIN3_At	Dispersed
CotAD_68019	GhPIN3_Dt	Dispersed
CotAD_58548	GhPIN9_At	Dispersed

Table S3 | A list of primers used in qRT-PCR experiments.

Gene ID	Sense primer sequence	Antisense primer sequence
GhPIN1b_Dt	ACCCATTATCCGGTTCCTAA	TATGCCCTTCAACTTTCCCT
GhPIN1c_Dt	ATGTTTACGGTTTGTCTGCC	GTCGGATACAGGAGAAGCAC
GhPIN1d_At	GTAATAAGGCAGCAACGAAC	TGAATAAGCCAAGGCTAAAC
GhPIN6_Dt	GGGAGCAAACAAGAAATGCC	ACTGAACATTGCCATCCCAA
GhPIN8a_At	TGTTGGCTATGGGTCTCACT	CGATAACGAAACGTCTCCTC
GhPIN8a_Dt	AGTATTCGCCTTCGTTGTTT	GCTTCCGTGTCCTCTATGTC
GhPIN1a_Dt	TAATCAAGAGGAGTTTATGG	AAGCCTTGTCATTACACTCG
GhPIN2_Dt	TATTATCCGATGCTGGTCTG	GTGCTAAGTATGTCGGGATG
GhPIN8_Dt	CAACCCTTACAAGATGAACC	GTAGAACGACAATCTGAGCC
GhPIN2_At	TTTTCCTATGGCAAGCCTTCA	CGTATATGTCCTTTCCAGTGATCAT
GhPIN1d_Dt	CCGTAATACTTTCCGTGATG	TGTTGCCAATACCACTCCTA
GhPIN8_At	CAACCCTTACAAGATGAACC	GTAGAACGACAATCTGAGCC
GhPIN9_At	GGTTGAGAGTGTGAAGCAAG	CATCCCAAGACCTGCACT
GhPIN3_Dt	GGTGGGTATTGGTTGTGATAG	AAGATAACCGCAGTGCTAAGA
GhPIN6_At	GGGAGCAAACAAGAAATGCC	CTGAACATTGCCATCCCAAG
GhPIN1c_At	ATGTTTACGGTTTGTCTGCC	GGGAGACAGCCACTCTAACC
GhPIN3_At	ATCCGTCACGAAGAACGCTAA	TGCTCAGACCGTCCAGATTG
GaPIN1a	GTGTTACCGACCAGAAAGAA	AAGCCTTGTCATTACACTCG
GaPIN1b	AGGGAAAGTTGAAGGGCATAG	CACCTGTTGGAGGCATTGTA
GaPIN1c	TATGGAGCAAACGAGCAGAA	TTCGTGGTTGTTGGTCATTT
GaPIN1d	CGGAATCCCAACACTTACTC	AGTCCCAATAGAATGTAGTAAACC
GaPIN5a	ACCGCCTCGTCTGCTATTTC	TCTCCTGTTCTCCGCCATCT
GaPIN6	CATCCTCCATTTGCCTCACC	GCATCCGACGAAGCATACCC
GaPIN8	TATCGGTGAAATGGTGGAAG	GCAAGAAATCGGCAAGTATG
GaPIN2	ACTCTTCTAAACCTACCCATCA	TATCTCCAAATCCTTGTCCC
GaPIN9	TGATTCAAGTTGTGGTGCTG	TGGTTAGATGATGTTGTGGC
GaPIN5b	AACCGCCTCGTCTGCTATTT	TCTCCTGTTCTCCGCCATCT
GaPIN3	AATCCCAATACATATTCCAGTC	AAAGATAACCGCAGTGCTAA
GrPIN3	AAGTTGGGGTCTAGTTCAACG	AAGCGGCAGCCATGACAGCC
GrPIN6	AATTAGCTTTAGAGACAACACC	TTGTTCCACATGCAATAATCC
GrPIN1c	TTGGGAACCGAGGATTGGAAC	ACCCATGGCAAAAGCTGCAACG
GrPIN1b	AACACGAAGGTGACAAGTTG	AATGCCATGAACAGACCGAG
GrPIN1d	ATGCCTCCGGCTAGTGTTATG	GGCACGATCCCTTGTGGAAG
GrPIN1a	AACCATGCCACCAACGAGTG	AAGACCAACAGCAATGGAAGC
GrPIN2	CATGGTTTGGAGGAAGCTCAT	AAGGGCAGCCTGAACGATAGCG
GrPIN5	AACTCCTATGCTTGTGTTATC	GAAGATGAAGGAGGTAATCG
GrPIN8	CCACTCTTTTGGCTCAGATTG	ACCTAAGCTGAACATTGCCAT
UBQ7	GGCATTCCACCTGACCAACAA	CCGCATTAGGGCACTCTTTTC
